# Supplementary material for: Noncoding mutations target cis-regulatory elements of the FOXA1 plexus in prostate cancer
Source: Nat Commun. 2020 Jan 23;11:441. doi: 10.1038/s41467-020-14318-9 (PMC6978390; doi:10.1038/s41467-020-14318-9)
Supplement: Supplementary file 1 — Supplementary Information [file 41467_2020_14318_MOESM1_ESM.pdf]

Noncoding mutations target *cis*-regulatory elements of the *FOXA1* plexus in prostate cancer

(Zhou *et al*.)

Supplementary Figure 1 - *FOXA1* mRNA expression in prostate tumors.

Supplementary Figure 2 - *FOXA1* mRNA expression across prostate cancer cell lines.

Supplementary Figure 3 - Essentiality of *FOXA1* across cancer cell lines of various cancer types.

Supplementary Figure 4 - Visualization of the functional annotation of the six *FOXA1* CREs.

Supplementary Figure 5 - Validation of clonal Cas9-mediated deletions of CREs.

Supplementary Figure 6 - Genome editing efficiency (%) is inversely correlated with *FOXA1* mRNA expression.

Supplementary Figure 7 - Intra-TAD genes and *FOXA1* downstream genes are significantly changed upon deletion of CREs.

Supplementary Figure 8 - Validation of transient Cas9-mediated single deletion of CREs.

Supplementary Figure 9 - Validation of transient Cas9-mediated double deletion of CREs.

Supplementary Figure 10 - Comparison of *FOXA1* mRNA expression upon double versus single deletion of CRE(s).

Supplementary Figure 11 - Validation of Cas9-mediated deletion of CREs from lentiviral system expressing both Cas9 protein and gRNA for cell proliferation assays.

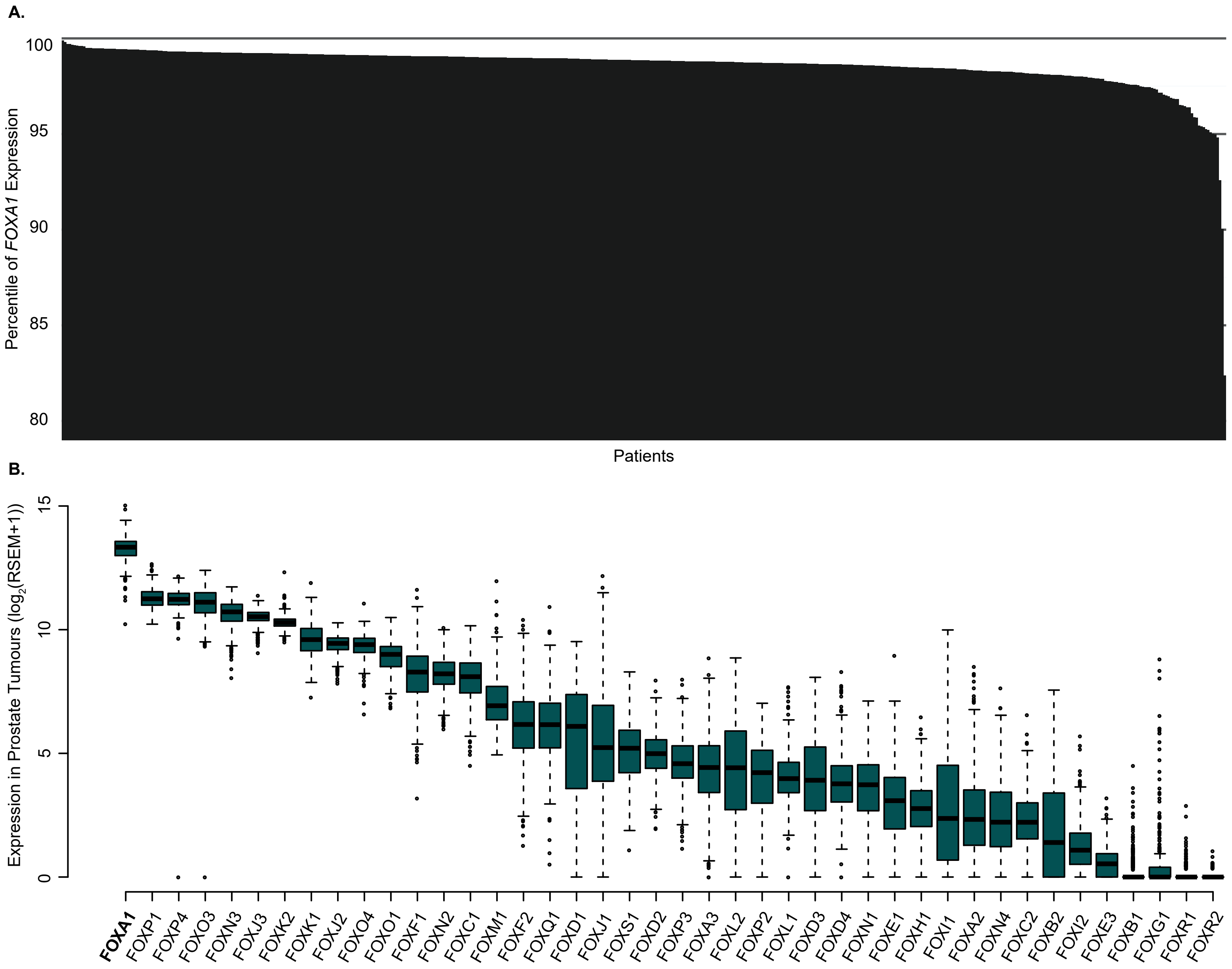

Supplementary Figure 1 - *FOXA1* mRNA expression in prostate tumors. **A.** The ranking of *FOXA1* mRNA expression across 497 primary prostate tumors profiled in TCGA. **B.** mRNA expression of all genes coding for FOX transcription factors across 497 primary prostate tumors profiled in TCGA.

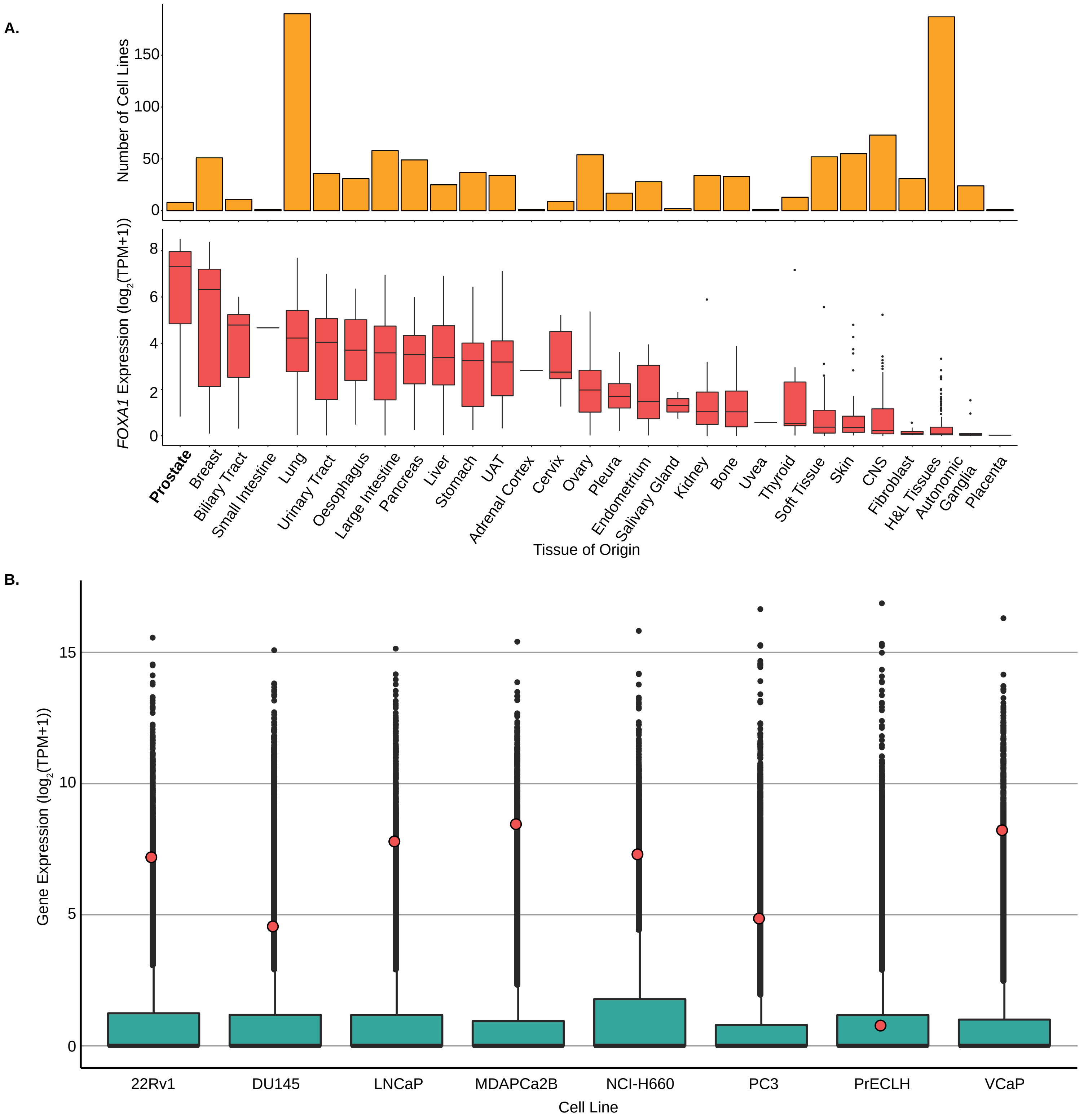

Supplementary Figure 2 - *FOXA1* mRNA expression across prostate cancer cell lines. **A.** *FOXA1* mRNA expression across all cancer cell lines from DEPMAP, profiled by RNA-seq (See Methods). UAT = Upper Aerodigestive Tract, CNS = Central Nervous System, H&L Tissues = Hematopoietic and Lymphoid Tissues. **B.** *FOXA1* mRNA expression across eight prostate cancer cell lines from DEPMAP, profiled by RNA-seq (See Methods). Red dot indicates *FOXA1*.

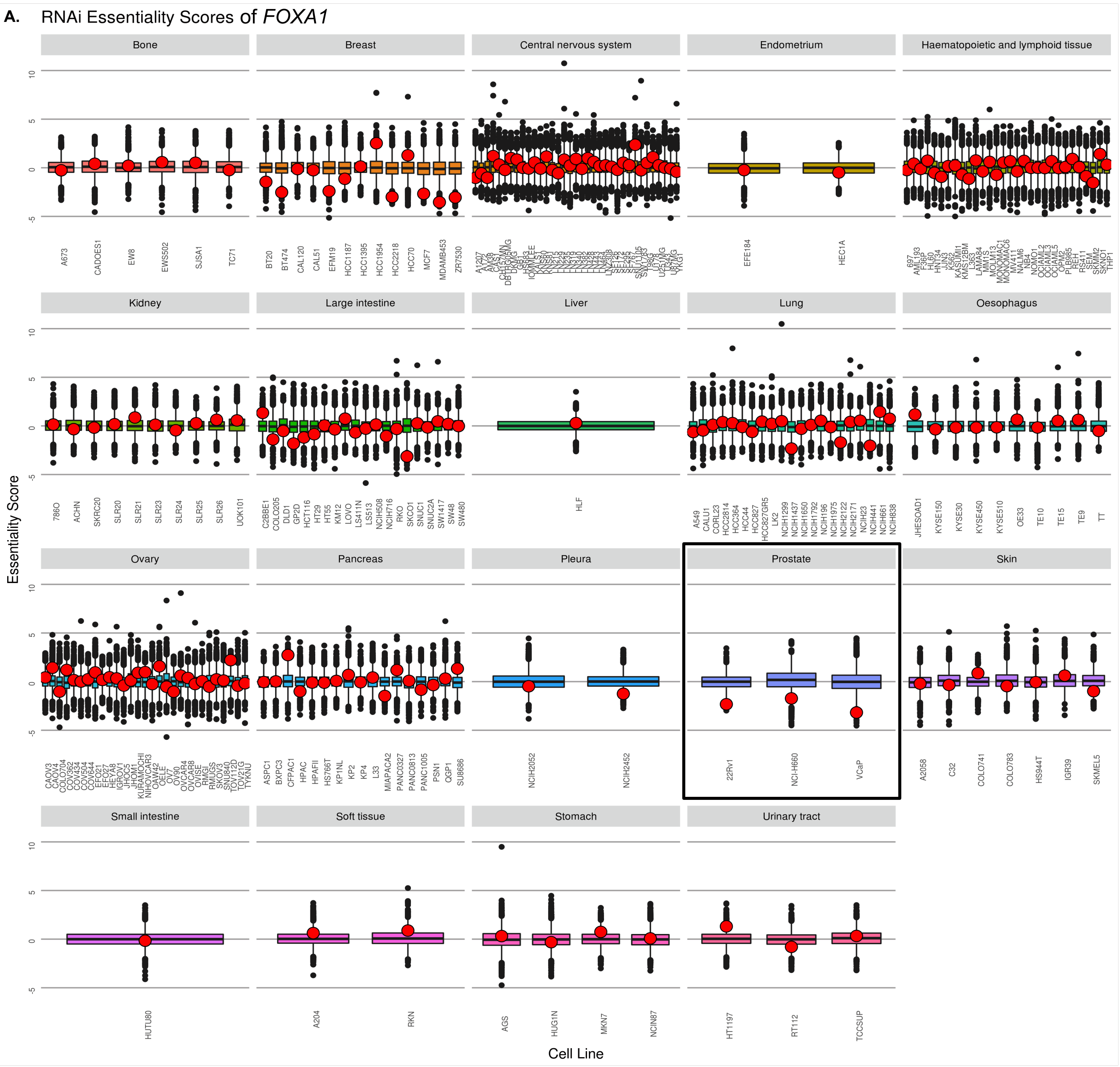

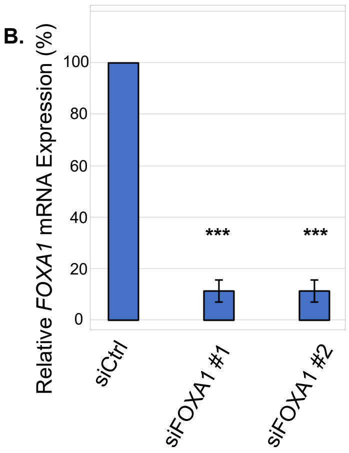

Supplementary Figure 3 - Essentiality of *FOXA1* across cancer cell lines of various cancer types. **A.** Gene essentiality screen mediated through shRNA/ RNAi across various cancer cell lines (n = 707). Higher score indicates less essential, and lower score indicates more essential for cell proliferation. Red dot indicates *FOXA1*. **B.** *FOXA1* mRNA expression normalized to housekeeping TBP mRNA expression upon siRNA-mediated knockdown, five days post-transfection (n=3 independent experiments). Error bars indicate ± s.d, Student's t-test, *** $p<0.001$.

Supplementary Figure 4

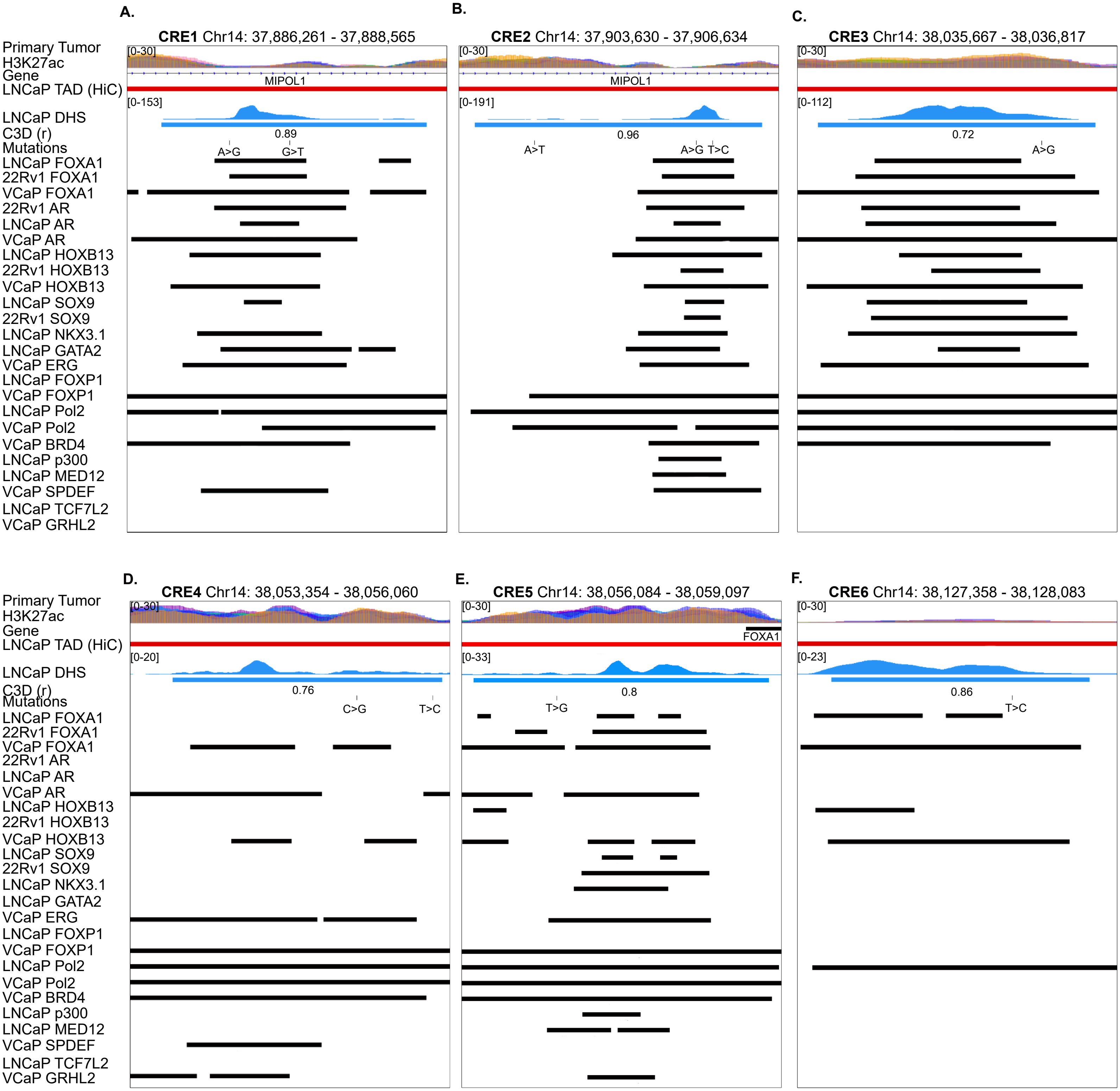

Supplementary Figure 4 - Visualization of the functional annotation of the six *FOXA1* CREs. **A-F.** Visualization of Functional annotation of the six *FOXA1* CREs using public and inhouse ChIP-seq datasets.

Supplementary Figure 5

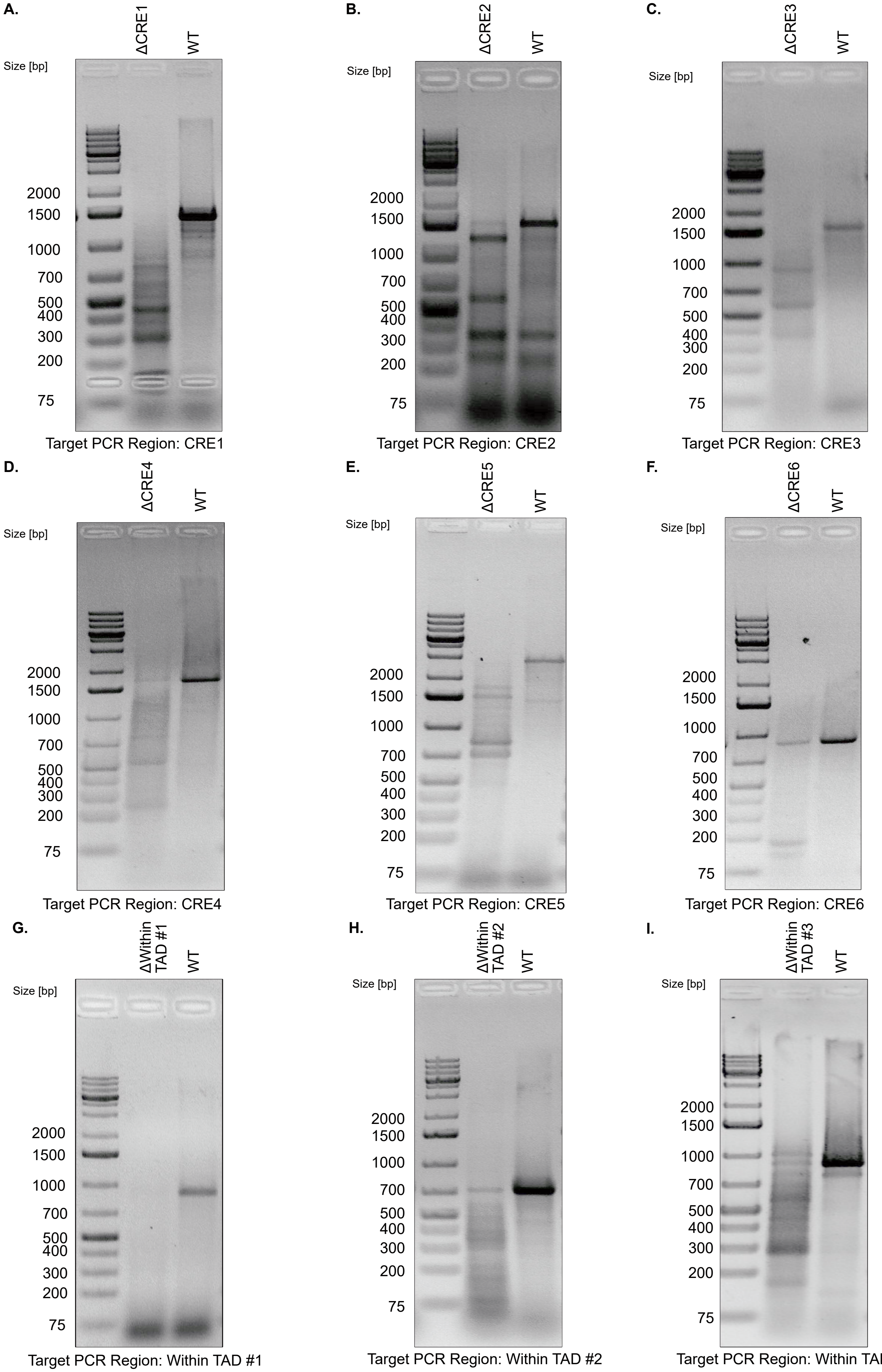

Supplementary Figure 5 - Validation of clonal Cas9-mediated deletions of CREs. **A-F.** Representative agarose gels from LNCaP clonal CRISPR/Cas9-mediated deletion product or wild-type (WT) product from PCR amplification of intended CRE, followed by T7 Endonuclease I assay.

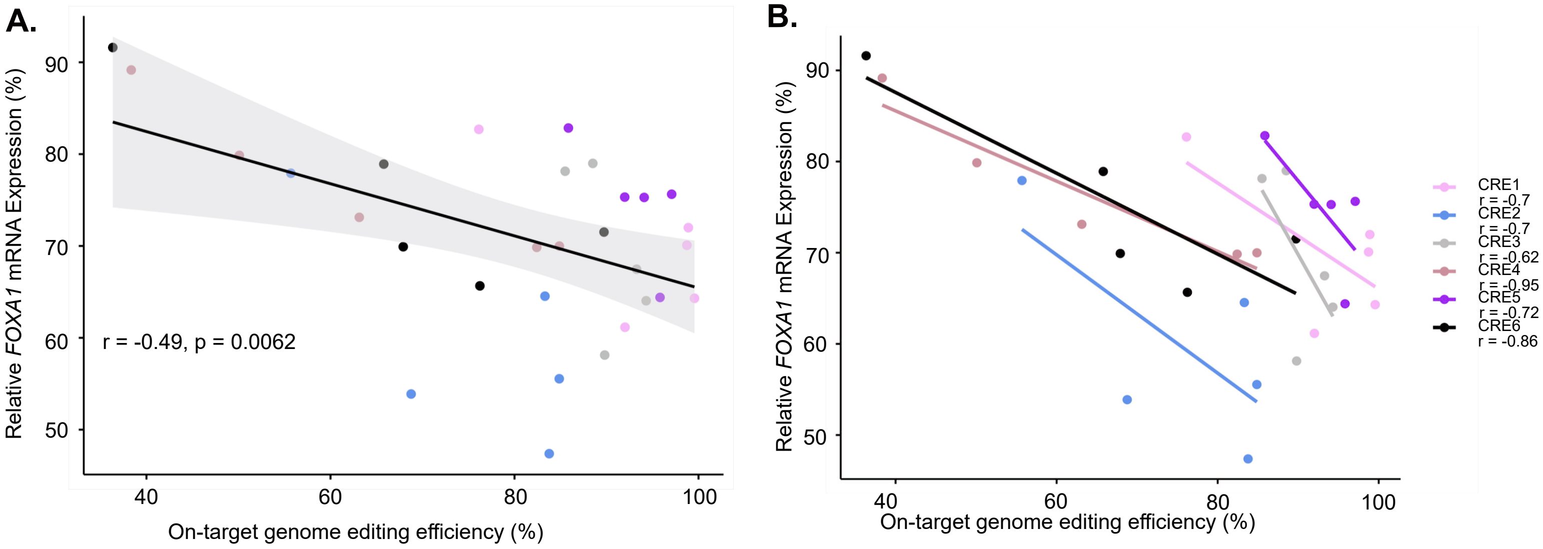

Supplementary Figure 6 - Genome editing efficiency (%) is inversely correlated with *FOXA1* mRNA expression. **A.** Pearson's correlation to investigate the relationship between genome editing efficiency mediated by CRISPR/Cas9 and *FOXA1* mRNA expression in LNCaP cells. The Pearson's correlation here is across all of the CREs. **B.** Pearson's correlation based on each individual CRE, correlation between genome editing efficiency mediated by CRISPR/Cas9 and *FOXA1* mRNA expression in LNCaP cells.

Supplementary Figure 7

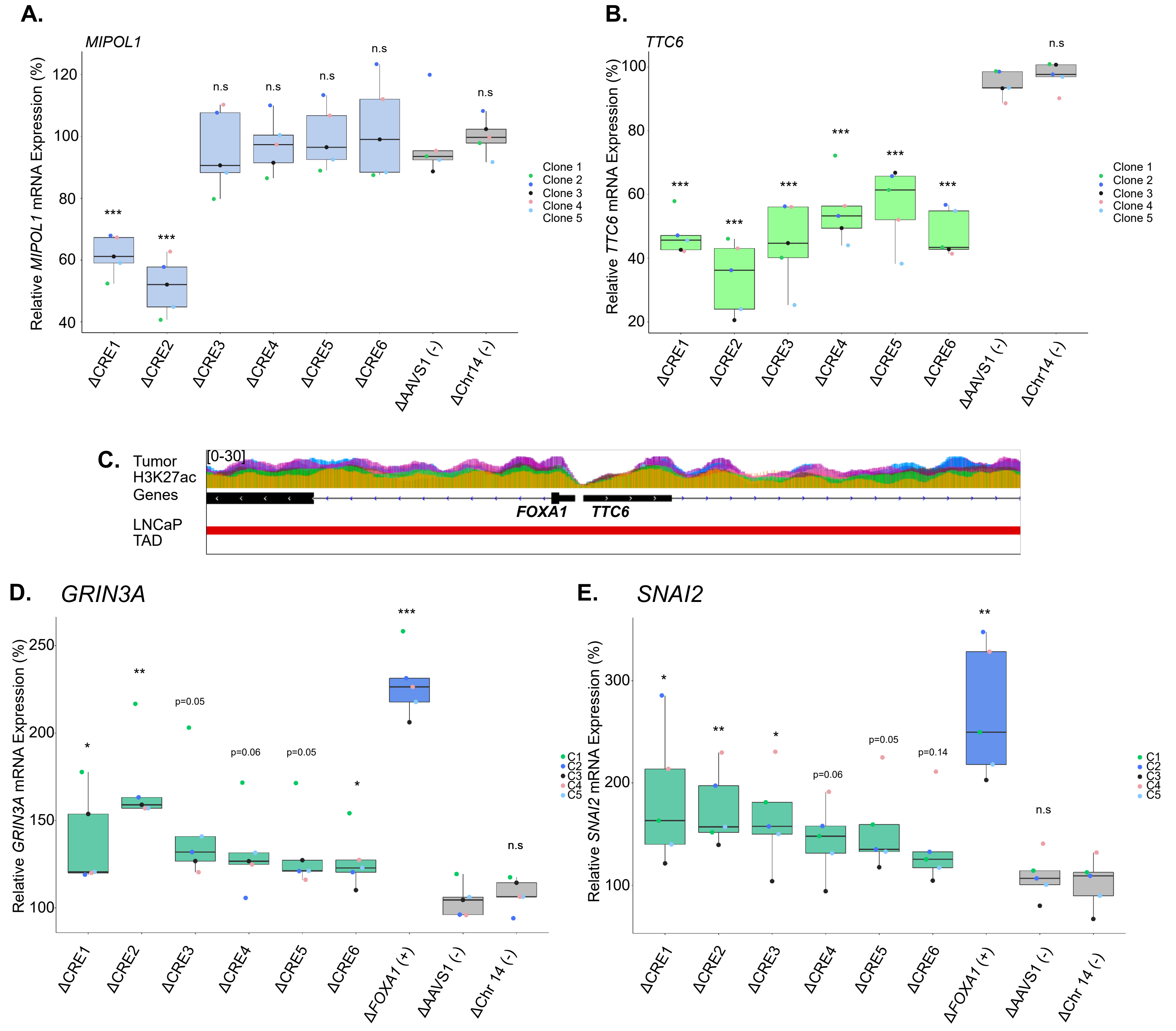

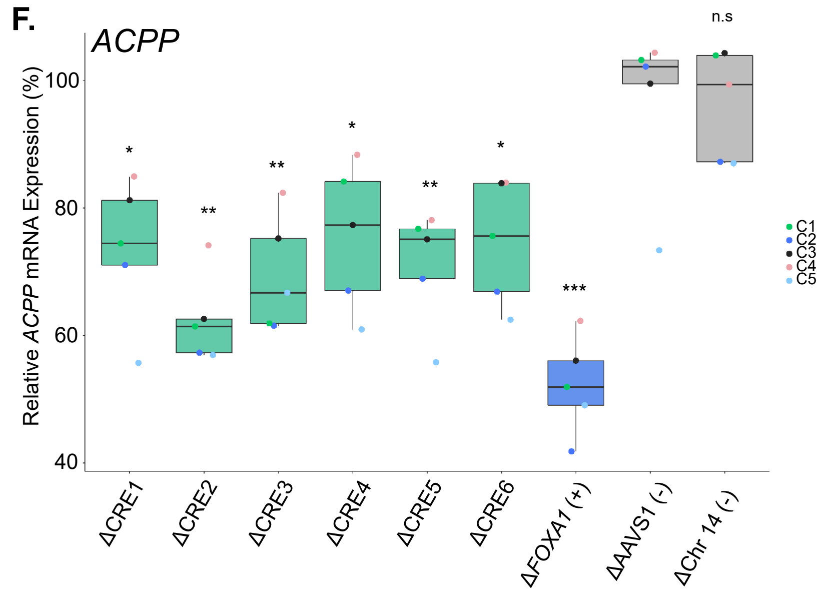

Supplementary Figure 7 - Intra-TAD genes and FOXA1 downstream genes are significantly changed upon deletion of CREs. **A.** *MIPOL1* mRNA expression normalized to housekeeping gene TBP upon deletion of each region of interest. **B.** *TTC6* mRNA expression normalized to housekeeping gene *TBP* upon deletion of each CRE. **C.** Zoom-in view of the *FOXA1* and *TTC6* locus. **D-F.** mRNA expression of *GRIN3A*, *SNAI2* and *ACPP* normalized to housekeeping gene TBP upon deletion of each region of interest. Δ indicates CRISPR/Cas9-mediated deletion (n=5 independent experiments, each dot represents an independent clone). Error bars indicate ± s.d. Student's t-test, * p<0.05, ** p<0.01, *** p<0.001.

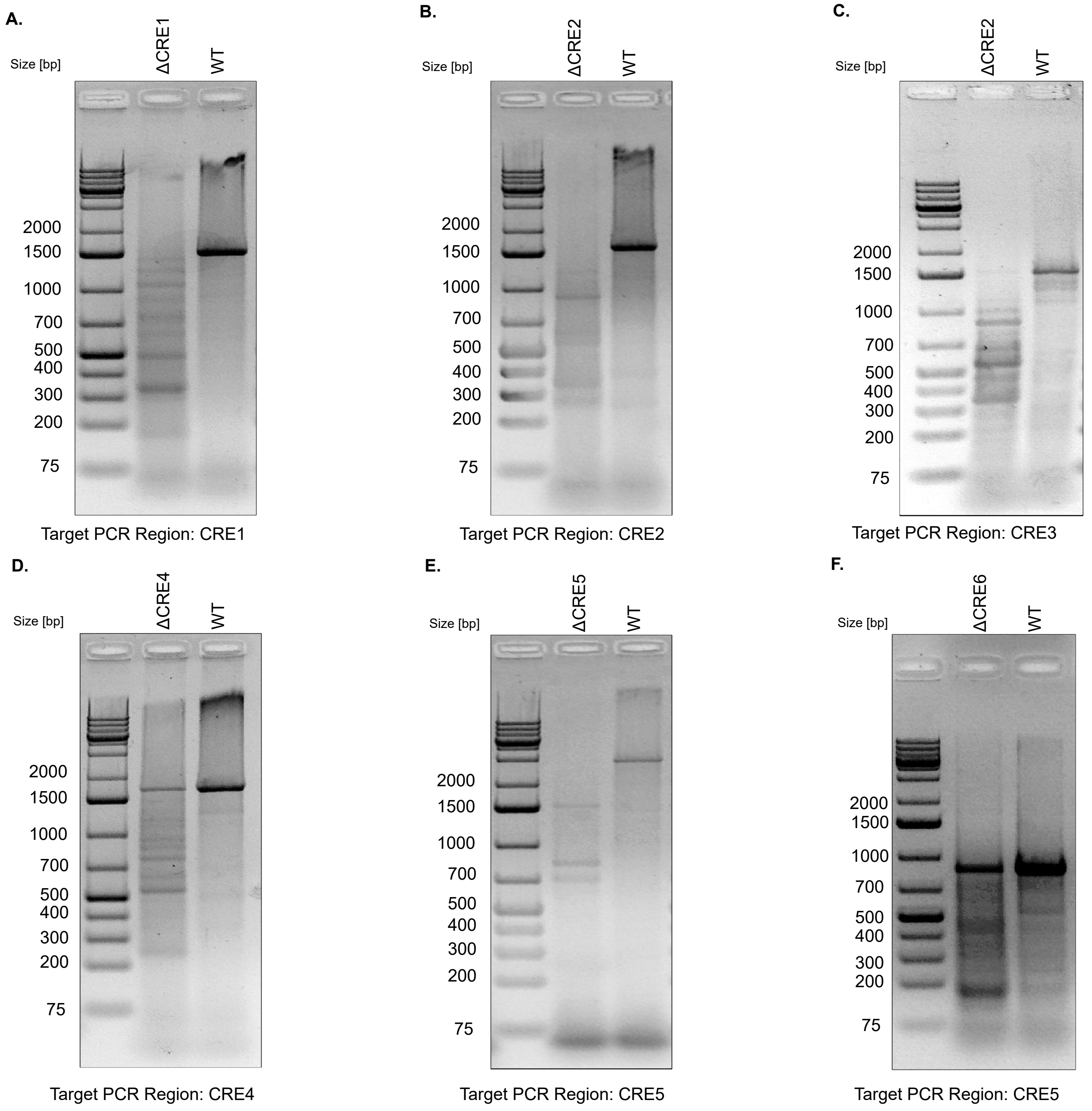

Supplementary Figure 8 - Validation of transient Cas9-mediated single deletion of CREs. **A-F.** Agarose gel of transient transfection RNP-based Cas9-mediated deletion product from PCR amplification of intended CRE followed by T7 Endonuclease I assay.

Supplementary Figure 9

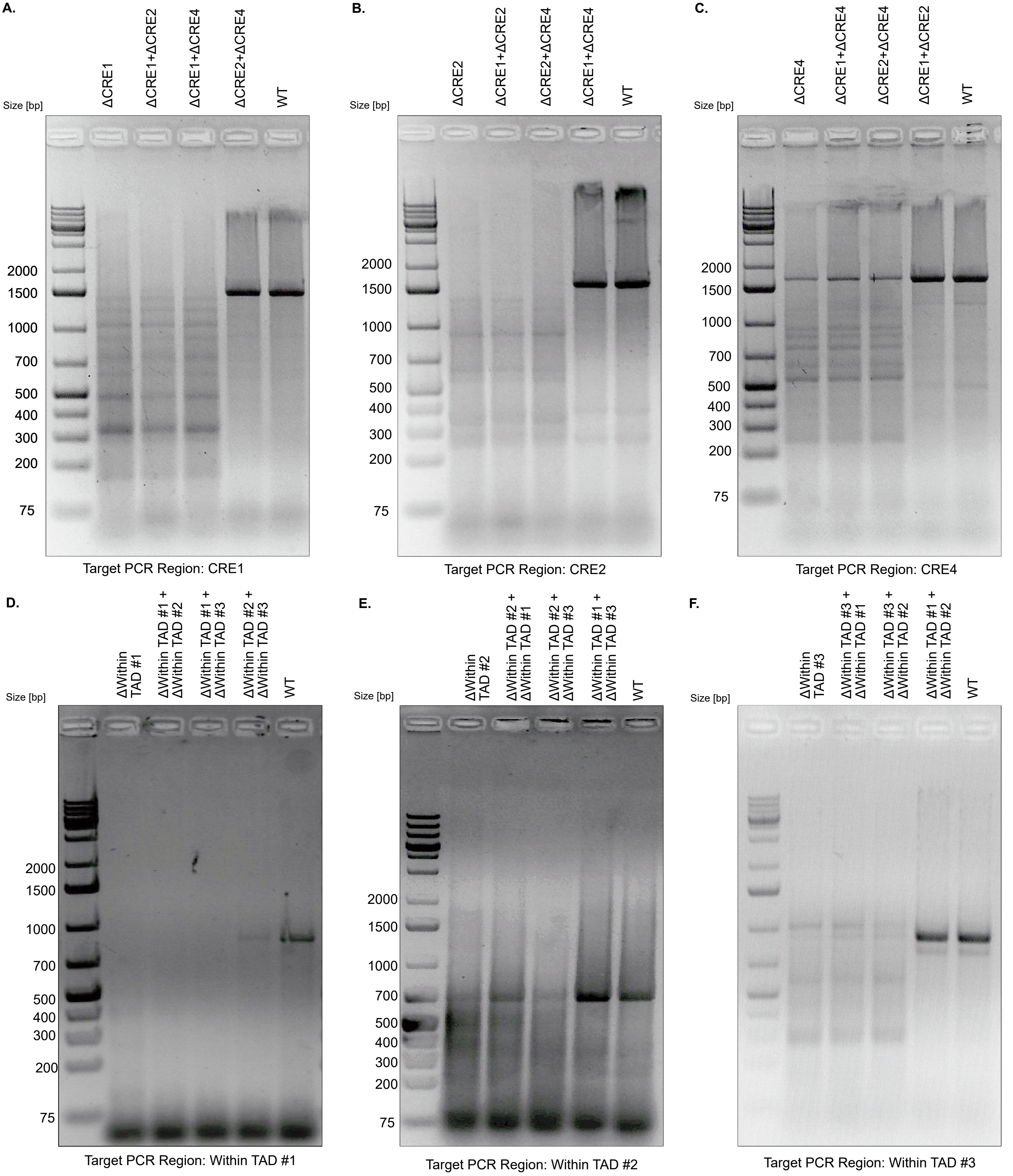

Supplementary Figure 9 - Validation of transient Cas9-mediated double deletion of CREs. **A-F.** Agarose gel of transient transfection RNP-based Cas9-mediated deletion product from PCR amplification of intended CREs followed by T7 Endonuclease I assay.

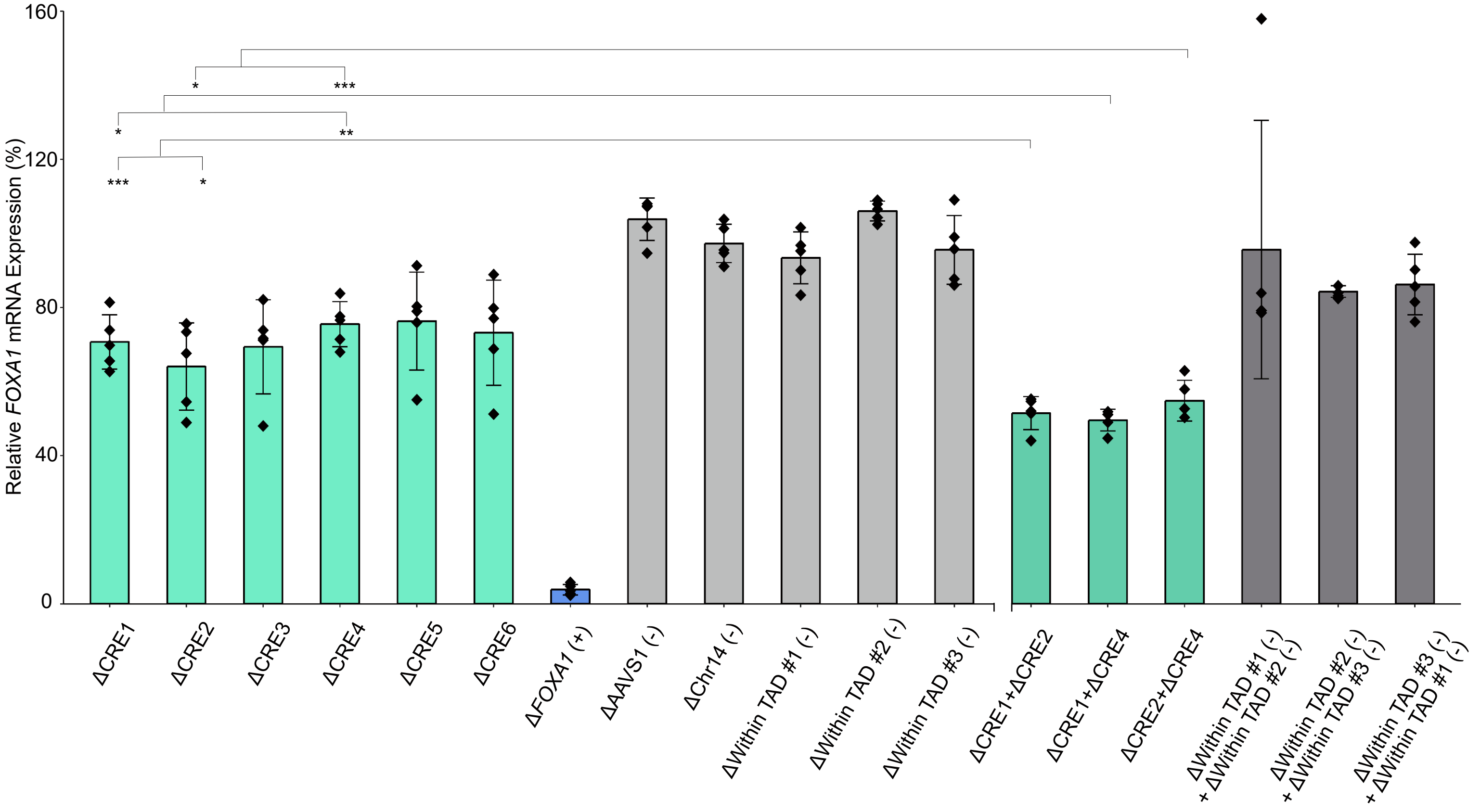

Supplementary Figure 10 - Comparison of *FOXA1* mRNA expression upon double versus single deletion of CRE(s). *FOXA1* mRNA expression normalized to housekeeping gene *TBP* upon single or double deletion of target CREs. Δ indicates CRISPR/Cas9-mediated deletion (n=5 independent experiments). Error bars indicate ± s.d., Student's t-test, * $p<0.05$, ** $p<0.01$, *** $p<0.001$.

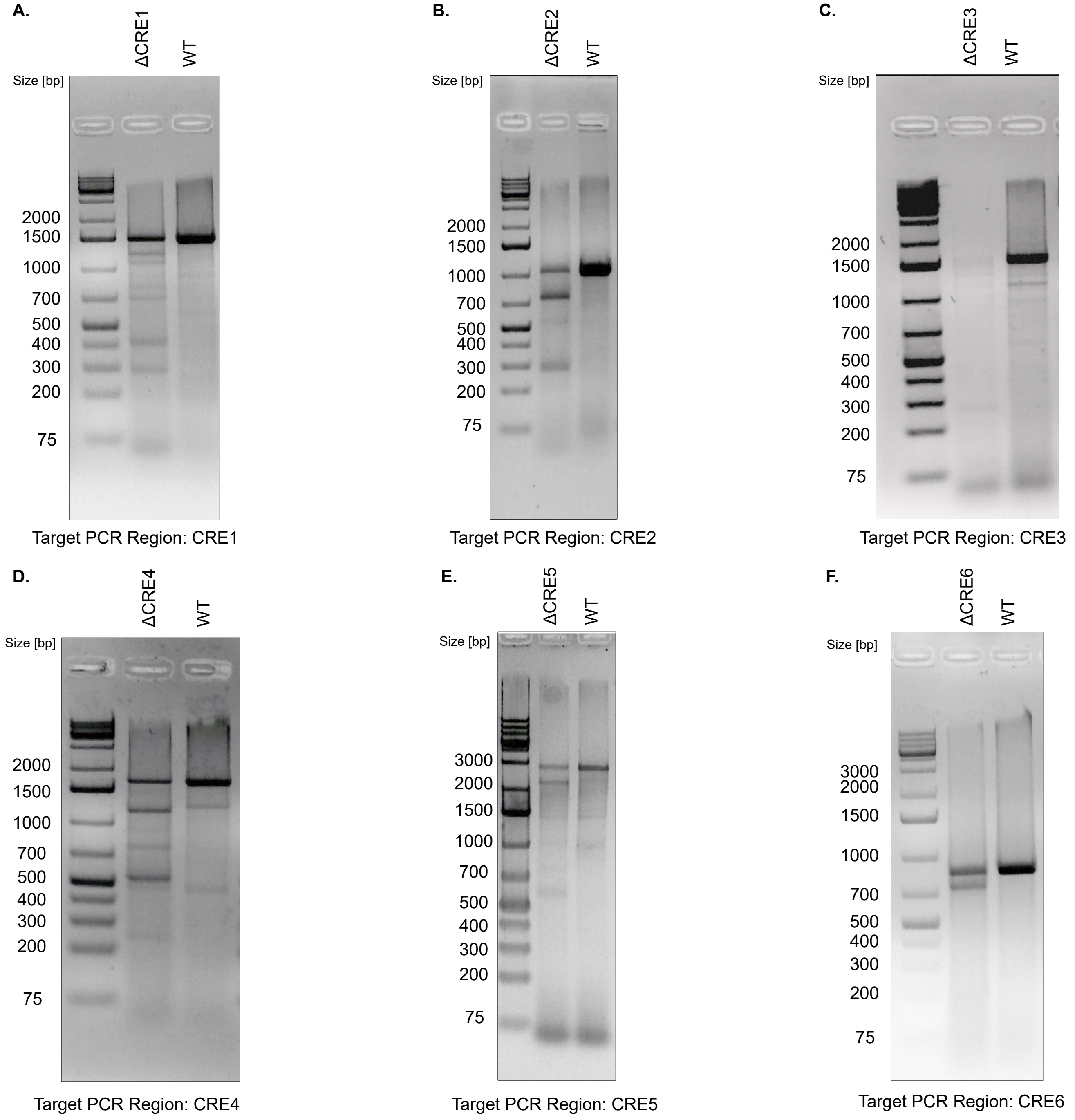

Supplementary Figure 11 - Validation of Cas9-mediated deletion of CREs from lentiviral system expressing both Cas9 protein and gRNA for cell proliferation assays. **A-F.** Agarose gel of lentiviral-based (expression of Cas9 protein and two gRNA) Cas9-mediated deletion product from PCR amplification of intended CREs followed by T7 Endonuclease I assay.
