# Supplementary material for: Noncoding mutations target cis-regulatory elements of the FOXA1 plexus in prostate cancer
Source: Nat Commun. 2020 Jan 23;11:441. doi: 10.1038/s41467-020-14318-9 (PMC6978390; doi:10.1038/s41467-020-14318-9)
Supplement: Supplementary file 3 — Description of Additional Supplementary Files [file 41467_2020_14318_MOESM3_ESM.pdf]

Description of Additional Supplementary Files

File name: Supplementary Data 1
Description: Tables of SNVs in the FOXA1 TAD, primers, gRNA, and Oligonucleotides
