# Supplementary material for: Noncoding mutations target cis-regulatory elements of the FOXA1 plexus in prostate cancer
Source: Nat Commun. 2020 Jan 23;11:441. doi: 10.1038/s41467-020-14318-9 (PMC6978390; doi:10.1038/s41467-020-14318-9)
Supplement: Supplementary file 4 — Supplementary Data 1 [file 41467_2020_14318_MOESM4_ESM.pdf]

**SNVs within TAD**

| Chromosome | Coordinate | SNV |
|---|---|---|
| chr14 | 37695514 | c>G |
| chr14 | 37700981 | a>G |
| chr14 | 37703714 | a>T |
| chr14 | 37715593 | C>T |
| chr14 | 37715710 | T>C |
| chr14 | 37730447 | A>G |
| chr14 | 37755665 | g>T |
| chr14 | 37762423 | A>G |
| chr14 | 37781081 | c>A |
| chr14 | 37785616 | c>T |
| chr14 | 37787646 | A>C |
| chr14 | 37803915 | a>G |
| chr14 | 37829761 | g>A |
| chr14 | 37857322 | G>C |
| chr14 | 37870712 | g>A |
| chr14 | 37884260 | T>C |
| chr14 | 37887005 | A>G |
| chr14 | 37887437 | G>T |
| chr14 | 37888682 | a>G |
| chr14 | 37888869 | g>T |
| chr14 | 37890288 | G>A |
| chr14 | 37903468 | t>G |
| chr14 | 37904343 | A>T |
| chr14 | 37905854 | A>G |
| chr14 | 37906009 | T>C |
| chr14 | 37918105 | G>A |
| chr14 | 37925787 | A>G |
| chr14 | 37941995 | g>A |
| chr14 | 37952831 | c>T |
| chr14 | 37966152 | a>T |
| chr14 | 37975348 | g>T |
| chr14 | 37997221 | C>G |
| chr14 | 38008477 | a>G |
| chr14 | 38012766 | g>A |
| chr14 | 38015049 | C>G |
| chr14 | 38022140 | c>T |
| chr14 | 38022335 | a>T |
| chr14 | 38036543 | A>G |
| chr14 | 38037962 | G>C |
| chr14 | 38051839 | G>T |

| | | |
|---|---|---|
| chr14 | 38055269 | C>G |
| chr14 | 38055908 | T>C |
| chr14 | 38056977 | T>G |
| chr14 | 38059153 | G>A |
| chr14 | 38060558 | C>T |
| chr14 | 38061208 | G>C |
| chr14 | 38061229 | A>C |
| chr14 | 38061231 | A>C |
| chr14 | 38061231 | A>G |
| chr14 | 38061231 | A>T |
| chr14 | 38061250 | G>A |
| chr14 | 38061294 | G>A |
| chr14 | 38061313 | C>T |
| chr14 | 38061317 | G>T |
| chr14 | 38061340 | A>C |
| chr14 | 38061528 | c>T |
| chr14 | 38061975 | G>T |
| chr14 | 38062840 | C>G |
| chr14 | 38072955 | C>A |
| chr14 | 38082628 | c>T |
| chr14 | 38097288 | g>A |
| chr14 | 38099831 | g>T |
| chr14 | 38103657 | g>A |
| chr14 | 38107989 | t>G |
| chr14 | 38109595 | T>C |
| chr14 | 38127842 | t>C |
| chr14 | 38145684 | a>G |
| chr14 | 38151460 | a>C |
| chr14 | 38152352 | T>G |
| chr14 | 38161158 | a>G |
| chr14 | 38165472 | A>T |
| chr14 | 38165665 | A>G |
| chr14 | 38178575 | t>A |
| chr14 | 38193969 | T>C |
| chr14 | 38197633 | a>C |

**Guide RNA for Clonal and Transient CRISPR/Cas9 and dCas9-KRAB experiments**

| Target | gRNA for Cas9 targeting | Location (Hg19) |
|---|---|---|
| CRE1_1 | GTGGAGGTTTAATTGAAACC | chr14:37886718-37886737 |
| CRE1_2 | AACATGACCATAGACCTACT | chr14:37886860-37886879 |
| CRE1_3 | CCAATTTCCATTCATGGTCT | chr14:37887321-37887340 |
| CRE1_4 | ACCCATTTTTCAATCACGTA | chr14:37887646-37887665 |
| CRE1_5 | TATTGAAATGTGGTCTTCTA | chr14:37887034-37887053 |
| CRE1_6 | CATAATTCCAGGTGACATAA | chr14:37887749-37887768 |
| CRE2_1 | TGACTATAGCTGGTGTTCAC | chr14:37905489-37905508 |
| CRE2_2 | CAGGTGAATAAAGTCTACTC | chr14:37905215-37905234 |
| CRE2_3 | ATTTACTAATGCATGTGTCA | chr14:37905617-37905636 |
| CRE2_4 | AACTTAACAGCAGGTTGCCT | chr14:37904850-37904869 |
| CRE2_5 | GCATTAAGCTTAATTTGTGC | chr14:37905060-37905079 |
| CRE2_6 | CAGAAGCAGGATTCTGTGAC | chr14:37905425-37905444 |
| CRE2_7 | TGGCAGTACAGTCAGATATC | chr14:37905915-37905934 |
| CRE2_8 | CTTAGGTTTGGAATAGTCAG | chr14:37906218-37906237 |
| CRE3_1 | CACTATTTCTCGATCCAAAC | chr14:38035926-38035945 |
| CRE3_2 | GCGGTAAATACTAGAAATTT | chr14:38036041-38036060 |
| CRE3_3 | GTTTCTGGGAACCTGTTAGT | chr14:38036102-38036121 |
| CRE3_4 | GAGTCCGCCTTATCTCCACA | chr14:38036347-38036366 |
| CRE3_5 | GTCCTTTAAGACTCCATATG | chr14:38036460-38036479 |
| CRE3_6 | CTTCTATCCCCTTATCTAAA | chr14:38036572-38036591 |
| CRE4_1 | CCTTCGTGCGACACGTAGTT | chr14:38053998-38054017 |
| CRE4_2 | TGATGCTAATGCTCGGTCCT | chr14:38054179-38054198 |
| CRE4_3 | TAACCCGGATCCTTAGCGGA | chr14:38054385-38054404 |
| CRE4_4 | CGAGGCCCTGCGCTACAATA | chr14:38054499-38054518 |
| CRE4_5 | CGCAGCGGTAGTTGGCGCCC | chr14:38054577-38054596 |
| CRE4_6 | AACTGACCCGGGATATGAGC | chr14:38054901-38054920 |
| CRE5_1 | CACAGGTGTTCGGAAGTAGA | chr14:38056803-38056822 |
| CRE5_2 | AAATGTTTGCTCGGTAGCGT | chr14:38057009-38057028 |
| CRE5_3 | TCGCCAGCTCCCGAAGACCA | chr14:38057208-38057227 |
| CRE5_4 | CCGCAGGAGCCGTAACGAGG | chr14:38057452-38057471 |
| CRE5_5 | CCCGCGCGCGTCCTAACGCG | chr14:38057639-38057658 |
| CRE5_6 | CTTCCGAGGTCTCCAAATTA | chr14:38058088-38058107 |
| CRE6_1 | AGTTTAAGCTTTGTTGAACC | chr14:38127236-38127255 |
| CRE6_2 | GTTAGTCATTTTATGGGATC | chr14:38127547-38127566 |
| CRE6_3 | ACATTTTTGTCTGACCTTGC | chr14:38127651-38127670 |
| CRE6_4 | ACACACATGGATGTAGTGTA | chr14:38127732-38127751 |
| CRE6_5 | TTCAGACATTTTACTTACCC | chr14:38127948-38127967 |
| CRE6_6 | GTTGGAGCTAAATTACACAA | chr14:38128193-38128212 |
| FOXA1(+)_1 | TCTTTGTGCGGCGGACAAAT | chr14:38064525-38064544 |
| FOXA1(+)_2 | GAGTTCAATCCAGTATCGCC | chr14:38066997-38067016 |

| | | |
|---|---|---|
| FOXA1(+)_3 | CGCAGTACCTGAGCGGCACT | chr14:38067708-38067727 |
| FOXA1(+)_4 | ACGTCTGCGAATTAAACGGT | chr14:38063687-38063706 |
| FOXA1(+)_5 | CCCGACTCTCGCAGCCGGAG | chr14:38063887-38063906 |
| FOXA1(+)_6 | GTAGTAGCTGTTCCAGTCGC | chr14:38064121-38064140 |
| AAVS1 (-)_1 | ATTCCCAGGGCCGGTTAATG | chr19:55627186-55627205 |
| AAVS1 (-)_2 | GTCCCCTCCACCCCACAGTG | chr19:55627139-55627158 |
| AAVS1 (-)_3 | GGGGCCACTAGGGACAGGAT | chr19:55627120-55627139 |
| AAVS1 (-)_4 | ACTAGGAAGGAGGAGGCCTA | chr19:55627077-55627096 |
| AAVS1 (-)_5 | CGTGGGGTACCCTAAGAACT | chr19:55625766-55625785 |
| AAVS1 (-)_6 | GATTCCCTTCTCAGGTTACG | chr19:55626035-55626054 |
| chr14 different TAD non-targeting (-)_1 | AAACGTCACTAATGTTGGGG | chr14:30211476-30211495 |
| chr14 different TAD non-targeting (-)_2 | AAGAGCCCTCACCCCCATGA | chr14:30212122-30212141 |
| chr14 different TAD non-targeting (-)_3 | GCGAGTCCAAAATCTGCTAT | chr14:30211558-30211577 |
| chr14 different TAD non-targeting (-)_4 | GTATTTATCTAGCTCTCAGT | chr14:30212024-30212043 |
| chr14 different TAD non-targeting (-)_5 | GCCGTACAGCATTTTACAAG | chr14:30211388-30211407 |
| chr14 different TAD non-targeting (-)_6 | GCATATCTCAGACCTTCATG | chr14:30212137-30212156 |
| Within_TAD_Neg1_1 | CATATCAGCTGTAGTGTATG | chr14:37812350-37812369 |
| Within_TAD_Neg1_2 | TATTCCAAGTTATTGTAATG | chr14:37812510-37812529 |
| Within_TAD_Neg1_3 | TTCACATTGACACCAAAGTT | chr14:37812661-37812680 |
| Within_TAD_Neg1_4 | AGAGTTGTATCACATCCAAG | chr14:37812733-37812752 |
| Within_TAD_Neg1_5 | TAAAAACTTAGACGATGTTA | chr14:37812833-37812852 |
| Within_TAD_Neg1_6 | GCTGGTATCATGAATCCTTA | chr14:37813115-37813134 |
| Within_TAD_Neg2_1 | AGCCACCCCACAGTTGTTAA | chr14:38151715-38151734 |
| Within_TAD_Neg2_2 | GCATGCTTGAAACTAAGCAT | chr14:38151841-38151860 |
| Within_TAD_Neg2_3 | TTTCCAACAGAGCGTGCATA | chr14:38151953-38151972 |
| Within_TAD_Neg2_4 | TACCGTTAAAGACACCCACT | chr14:38152153-38152172 |
| Within_TAD_Neg2_5 | CCATACGTTGGTCCTTGCCT | chr14:38152244-38152263 |
| Within_TAD_Neg2_6 | GAAGTGCAAGTTGCCCACTT | chr14:38152312-38152331 |
| Within_TAD_Neg3_1 | CATTTTAGCTAAGGACTTGC | chr14:37875337-37875356 |
| Within_TAD_Neg3_2 | GTATAGAAGAAATTCTACCC | chr14:37875447-37875466 |
| Within_TAD_Neg3_3 | GTTTGGTTCAGCATGTGTTC | chr14:37875530-37875549 |
| Within_TAD_Neg3_4 | TTACGACTCAAGTGTAAAGG | chr14:37875669-37875688 |
| Within_TAD_Neg3_5 | GTACCAGAGAGCTTAGTGAA | chr14:37875775-37875794 |
| Within_TAD_Neg3_6 | TACCAAGCCAACTGTTCTTG | chr14:37875913-37875932 |

**CRISPR/Cas9 Deletion PCR Validation Primers**

| Region | | Sequence |
|---|---|---|
| CRE1 | Forward | GACTGAGGAACCTCATTATCTCTG |
| | Reverse Complement | GCCCAAACTTTCTGCCTATAATG |
| CRE2_Lenti | Forward | GCAGGTTGCCTGGGAAGTGAG |
| | Reverse Complement | CTGCCAGTGCCACCCAGTC |
| CRE2_EverythingElse | Forward | GCAGGTTGCCTGGGAAGTG |
| | Reverse Complement | AGCCAGTCCCTATCCCTAGGC |
| CRE3 | Forward | GCACATGGAAAGGGGATGTG |
| | Reverse Complement | CAACCAGGCTGTTATGCTGG |
| CRE4 | Forward | CTGGCGTAGCGCAGGAGATC |
| | Reverse Complement | CACTCCTCCCCCTTGCAGTC |
| CRE5 | Forward | GGCATGCTCTTAACTCCATTTAGTTGC |
| | Reverse Complement | GCCTTCTGTGTTTCCTTTGAGCC |
| CRE6 | Forward | GGTTCATTTTAGAGATGCATTTGTTC |
| | Reverse Complement | CCCCTGAACCTAAAATAAAAAAATTTTTAAAG |
| TAD1- | Forward | GTG CTG ATA TGT TGC CTA ATG G |
| | Reverse Complement | GCAGCAGGCTGAAAATACC |
| TAD2- | Forward | CAT AGT ATT TGG TAT TGT ATG CCA TC |
| | Reverse Complement | GGTTGACACTAGAAACCCTC |
| TAD3- | Forward | CTT TCT TTA TAG CCT ATG CAC C |
| | Reverse Complement | CACTGACTATTTCACTGGTTTC |

**RT-PCR mRNA Expression Primers**

| Gene | Primer Forward | Primer Reverse |
|---|---|---|
| FOXA1 | GAA GAT GGA AGG GCA TGA AA | GCC TGA GTT CAT GTT GCT GA |
| TBP | TGC ACA GGA GCC AAG AGT GAA | CAC ATC CAC AGC TCC CCA CCA |
| SNAI2 | ACGCCTCCAAAAAGCCAAAC | ACTCACTCGCCCCAAAGATG |
| ACPP | CTTTCAGGAACTGCCCTCGT | GGTGCAGCCTCTTCTGGAAT |
| MIPOL1 | CCATCGCAAGGTTCTCAAGG | CCCTGGCCATTCTGTGTTCT |
| TTC6 | AAAGCTGTCCCTTTTGGGCT | CCTTGAGATTCTGCAACCTTGG |
| GRIN3A | CGGAGACTTTGCAAATGGGC | AGACCAAATCCAATGCACAGC |

**Guide RNA for lentiviral-based CRISPR/Cas9 deletion proliferation assays**

| **Target** | **gDNA for Cas9 targeting** | **Location (Hg19)** |
|---|---|---|
| CRE1 | AACATGACCATAGACCTACT | chr14:37886860-37886879 |
| CRE1 | ACCCATTTTTCAATCACGTA | chr14:37887646-37887665 |
| CRE2 | ATTTACTAATGCATGTGTCA | chr14:37905617-37905636 |
| CRE2 | GCATTAAGCTTAATTTGTGC | chr14:37905060-37905079 |
| CRE3 | TCTATGTTGTTATTAACTAG | chr14:38035682-38035701 |
| CRE3 | TTCCACTAGGAACAATAATG | chr14:38036962-38036981 |
| CRE4 | CCTTCGTGCGACACGTAGTT | chr14:38053998-38054017 |
| CRE4 | AACTGACCCGGGATATGAGC | chr14:38054901-38054920 |
| CRE5 | TGTGATCCCTCAATGTCAAC | chr14:38056269-38056288 |
| CRE5 | GAGTGGGGCGATCAAAGTAA | chr14:38058291-38058310 |

| | | |
|---|---|---|
| CRE6 | AGTTTAAGCTTTGTTGAACC | chr14:38127236-38127255 |
| CRE6 | TTCAGACATTTTACTTACCC | chr14:38127948-38127967 |
| FOXA1 Promoter (+) | TCTTTGTGCGGCGGACAAAT | chr14:38064525-38064544 |
| FOXA1 Promoter (+) | ACGTCTGCGAATTAAACGGT | chr14:38063687-38063706 |
| AAVS1 (-) | ATTCCCAGGGCCGGTTAATG | chr19:55627186-55627205 |
| AAVS1 (-) | ACTAGGAAGGAGGAGGCCTA | chr19:55627077-55627096 |
| chr14 non-targeting (-) | AAACGTCACTAATGTTGGGG | chr14:30211476-30211495 |
| chr14 non-targeting (-) | AAGAGCCCTCACCCCCATGA | chr14:30212122-30212141 |

**Primers for MAMA ChIP-qPCR**

| Name | Primer Sequence |
|---|---|
| Constant, pGL3 Promoter, after BamHI_ Reverse Complement | GAAGACAGTCATAAGTGCGG |
| 7005_A_F_MAMA | GGT TAC TCT GGA AAT AAC TCT ATT AAA A |
| 7005_G_F_MAMA | GGT TAC TCT GGA AAT AAC TCT ATT AATG |
| 7437_G_F_MAMA | CAA GAT CTC AAG GAG AGA TAA AAG TGG |
| 7437_T_F_MAMA | CAA GAT CTC AAG GAG AGA TAA AAG TCT |
| 5854_A_F_MAMA | GCC ACT GCT GTC ATA AAA AGC TTA |
| 5854_G_F_MAMA | GCC ACT GCT GTC ATA AAA AGC TAG |
| 6009_T_F_MAMA | CCCAAAATGATGAATGTTTACCTAT |
| 6009_C_F_MAMA | CCCAAAATGATGAATGTTTACCTTC |
| 6543_A_F_MAMA | GAACCAAGATCTGTGAAAGAAAAGTA |
| 6543_G_F_MAMA | GAACCAAGATCTGTGAAAGAAAAGAG |
| 7842_T_F_MAMA | CTTAATGAGTACATTGGGTTATTT |
| 7842_C_F_MAMA | CTTAATGAGTACATTGGGTTATGC |
| 5269_C_F_MAMA | CGC ATT CCA CCT GGA TTC |
| 5269_G_F_MAMA | CGC ATT CCA CCT GGA TTG |
| 5908_T_F_MAMA | GCTTTACCTTTCCAAATCAATTCTATTT |
| 5908_C_F_MAMA | GCTTTACCTTTCCAAATCAATTCTATGC |
| 6977_T_F_MAMA | CCC CTG TCC TAT GCT CAC T |
| 6977_G_F_MAMA | CCC CTG TCC TAT GCT CAAG |
| 4343_A_F_MAMA | CCAGATGTATGTTGTGATATTACTTATAA |
| 4343_T_F_MAMA | CCAGATGTATGTTGTGATATTACTTATCT |

**gBlock Sequences for Luciferase Assays**

| **Mutation** | **Wild Type or Mutant** | **gBlock Sequence** |
|---|---|---|

| | | |
|---|---|---|
| chr 14: 37887005 (A>G) | Wild Type | AACCAACGGATCCATCCTAGTAGGTCTATGGTCATGTTCTTTGAGAAATCATCTAACT<br>TGATGTATTGTTTGAAGATACCCAATTATATATTTTATAGATGTGTTTAATAGTGCTA<br>GCTTAATCATATATATTTATGGTTACTCTGGAAATAACTCTATTAAAATAACTATCAC<br>ACATATTAAATTATACCATATTGAAATGTGGTCTTCTATGGTAAAATAAGCAATGTTT<br>TCTGGATACTTGTTAGTTCCAACTTTCTTTCTAACCATCTATGAAACCTTGGACAATTT<br>CCAAATGTTTCCCTGAACATGTTGGATCCTAGAGAGGGG |
| | Mutant | AACCAACGGATCCATCCTAGTAGGTCTATGGTCATGTTCTTTGAGAAATCATCTAACT<br>TGATGTATTGTTTGAAGATACCCAATTATATATTTTATAGATGTGTTTAATAGTGCTA<br>GCTTAATCATATATATTTATGGTTACTCTGGAAATAACTCTATTAAAGTAACTATCAC<br>ACATATTAAATTATACCATATTGAAATGTGGTCTTCTATGGTAAAATAAGCAATGTTT<br>TCTGGATACTTGTTAGTTCCAACTTTCTTTCTAACCATCTATGAAACCTTGGACAATTT<br>CCAAATGTTTCCCTGAACATGTTGGATCCTAGAGAGGGG |
| chr 14: 37887437 (G>T) | Wild Type | AACCAACGGATCCCATAACATCAGATGTCTCTGTTCTAAGATAAAAACCAATTTCCAT<br>TCATGGTCTTGGAGTTACTAAAACAAGGCTTAACTGTATCAGACTATAACTATTAAAA<br>ACACATTTAAACCAAACATAACAAGATCTCAAGGAGAGATAAAAGTGGTTTATCCAC<br>AATACTGGTTAAGAATAGCGCTTAGTGATGTGAACAGTGTGGTCTTTTCTTTGTCATT<br>CTGAGTTACTAGATTAAGCTCCAGTGAAACAATGTAGTTCATTTCTGATACAGTCCCA<br>ACACAAATGGCTGTCCTATTTAGATGGATCCTAGAGAGGGG |
| | Mutant | AACCAACGGATCCCATAACATCAGATGTCTCTGTTCTAAGATAAAAACCAATTTCCAT<br>TCATGGTCTTGGAGTTACTAAAACAAGGCTTAACTGTATCAGACTATAACTATTAAAA<br>ACACATTTAAACCAAACATAACAAGATCTCAAGGAGAGATAAAAGTGTTTTATCCAC<br>AATACTGGTTAAGAATAGCGCTTAGTGATGTGAACAGTGTGGTCTTTTCTTTGTCATT<br>CTGAGTTACTAGATTAAGCTCCAGTGAAACAATGTAGTTCATTTCTGATACAGTCCCA<br>ACACAAATGGCTGTCCTATTTAGATGGATCCTAGAGAGGGG |
| chr 14: 37904343 (A>T) | Wild Type | AACCAACGGATCCAGGTAAAAATTACCCTCTCTAAAAGGGGCATGCACTTGCCATTT<br>CACCACACTTTCTATTCCACCTACTTTGTGAATTTAAAGTCATCTTCCTCAACCCTGTA<br>GGTATTTAATTTTATGACCCCAGATGTATGTTGTGATATTACTTATAAAATGGATGTT<br>TGTACACGTATACATATACTCTGGCCCCTGGCTACCTCTCTAACCTTATTTCTTAACAT<br>GTTTCTACTCTTGGTTCACTCCAGCCACACTAGGTTCCTTAATAACTCAAATATTCTAG<br>GTGTGCTCCCATCCCAGGGCCCGGATCCTAGAGAGGGG |
| | Mutant | AACCAACGGATCCAGGTAAAAATTACCCTCTCTAAAAGGGGCATGCACTTGCCATTT<br>CACCACACTTTCTATTCCACCTACTTTGTGAATTTAAAGTCATCTTCCTCAACCCTGTA<br>GGTATTTAATTTTATGACCCCAGATGTATGTTGTGATATTACTTATATAATGGATGTT<br>TGTACACGTATACATATACTCTGGCCCCTGGCTACCTCTCTAACCTTATTTCTTAACAT<br>GTTTCTACTCTTGGTTCACTCCAGCCACACTAGGTTCCTTAATAACTCAAATATTCTAG<br>GTGTGCTCCCATCCCAGGGCCCGGATCCTAGAGAGGGG |
| chr 14: 379 | Wild Type | AACCAACGGATCCATTGAAGACCTTTGTTCAGGAACATTCTGATTCATTAATTGCAAA<br>AACAATAAATGTTAAACATACAGTACAAGATCTAATAAACTGAGATCTTTAAGCTAGT |

| | | |
|---|---|---|
| 058<br>54<br>(A><br>G) | | TTTGATTTCTATAAACTTTCTAATGCCACTGCTGTCATAAAAAGCTT**A**GTAAATATTG<br>ACTGAGGATGATGATGACGATCCGTCAGTATTTTTAAGACTGGGTGGCACTGGCAG<br>TACAGTCAGATATCCGGGACTTAACATTGTTATTTTGCCATGAGGCCCTTCCACCTGG<br>CCCTATCCCAAAATGATGAATGTTTAGGATCCTAGAGAGGGG |
| | Mutant | AACCAACGGATCCATTGAAGACCTTTGTTCAGGAACATTCTGATTCATTAATTGCAAA<br>AACAATAAATGTTAAACATACAGTACAAGATCTAATAAACTGAGATCTTTAAGCTAGT<br>TTTGATTTCTATAAACTTTCTAATGCCACTGCTGTCATAAAAAGCTT**G**GTAAATATTG<br>ACTGAGGATGATGATGACGATCCGTCAGTATTTTTAAGACTGGGTGGCACTGGCAG<br>TACAGTCAGATATCCGGGACTTAACATTGTTATTTTGCCATGAGGCCCTTCCACCTGG<br>CCCTATCCCAAAATGATGAATGTTTAGGATCCTAGAGAGGGG |
| chr<br>14:<br>379<br>060<br>09<br>(T><br>C) | Wild<br>Type | AACCAACGGATCCATATTGACTGAGGATGATGATGACGATCCGTCAGTATTTTTAAG<br>ACTGGGTGGCACTGGCAGTACAGTCAGATATCCGGGACTTAACATTGTTATTTTGCC<br>ATGAGGCCCTTCCACCTGGCCCTATCCCAAAATGATGAATGTTTACCTA**T**AGTTAGG<br>TATTTAAATGTGTAAATATTCCAGATTCAATTATATGTGAGGTAGCTAGAGTTTTCAT<br>TCCTTAAATCATGAACTAGTTCTCTAAAGTTTAAATGATTTACAAGTCTGCAAGGGTC<br>AAAATTGACTTGACTGAATGTTTTCCCGGATCCTAGAGAGGGG |
| | Mutant | AACCAACGGATCCATATTGACTGAGGATGATGATGACGATCCGTCAGTATTTTTAAG<br>ACTGGGTGGCACTGGCAGTACAGTCAGATATCCGGGACTTAACATTGTTATTTTGCC<br>ATGAGGCCCTTCCACCTGGCCCTATCCCAAAATGATGAATGTTTACCTA**C**AGTTAGG<br>TATTTAAATGTGTAAATATTCCAGATTCAATTATATGTGAGGTAGCTAGAGTTTTCAT<br>TCCTTAAATCATGAACTAGTTCTCTAAAGTTTAAATGATTTACAAGTCTGCAAGGGTC<br>AAAATTGACTTGACTGAATGTTTTCCCGGATCCTAGAGAGGGG |
| chr<br>14:<br>380<br>365<br>43<br>(A><br>G) | Wild<br>Type | AACCAACGGATCCAATTTATTATGAAATGCTTTGTTTGTGTTAACATATATCTTCTCTG<br>GGAAGCTGGAAACAAAGGCATGTCCTTTAAGACTCCATATGGGGAAAACACATCCT<br>CCTTTGGAATTTAACCTTAATTTGAACCAAGATCTGTGAAAGAAAAGT**A**CTTTAGTG<br>TATTGTTCCCTTGCTCCACCCTTCTATCCCCTTATCTAAATGGAGTTACTGTTGCTTCG<br>TGTTTTTTTAACCCTTCCAATTCCAGTCCTTGCTTTCCTGAGTTTAAAATTTATCCTGG<br>GAAAGAAATATATTAAAATAATTGGGATCCTAGAGAGGGG |
| | Mutant | AACCAACGGATCCAATTTATTATGAAATGCTTTGTTTGTGTTAACATATATCTTCTCTG<br>GGAAGCTGGAAACAAAGGCATGTCCTTTAAGACTCCATATGGGGAAAACACATCCT<br>CCTTTGGAATTTAACCTTAATTTGAACCAAGATCTGTGAAAGAAAAGT**G**CTTTAGTG<br>TATTGTTCCCTTGCTCCACCCTTCTATCCCCTTATCTAAATGGAGTTACTGTTGCTTCG<br>TGTTTTTTTAACCCTTCCAATTCCAGTCCTTGCTTTCCTGAGTTTAAAATTTATCCTGG<br>GAAAGAAATATATTAAAATAATTGGGATCCTAGAGAGGGG |
| chr<br>14:<br>380<br>552<br>69 | Wild<br>Type | AACCAACGGATCCCCCTGATGTGTAATCTTGAAGGGGAGTTGAGAGACGTAAAAAG<br>TTAAACCAAGGCAACCTCACACTTAAATTCTGAGTCAGGCCTGCCGTTGGTGTCACT<br>GGCCTGGTTCTTGATTTCGCCAGTGACTTGACCCGCATTCCACCTGGATT**C**TGATGT<br>ATTCGAGCACGATTCTACTTAAGCCCTTTCCTTCCTGGATTTTGAGGGAGAATATCTT |

| | | |
|---|---|---|
| (C> | | GCCTCTGTCCTTTAGGTTGACTGGAACATAGAGAACCCCAAAAGATCACGGAGTGG |
| G) | | CACCCAGAAAAAGGAGGGCTCCTTATTTCCGGATCCTAGAGAGGGG |
| | | AACCAACGGATCCCCCTGATGTGTAATCTTGAAGGGGAGTTGAGAGACGTAAAAAG |
| | | TTAAACCAAGGCAACCTCACACTTAAATTCTGAGTCAGGCCTGCCGTTGGTGTCACT |
| | | GGCCTGGTTCTTGATTTCGCCAGTGACTTGACCCGCATTCCACCTGGATTGTGATGT |
| | | ATTCGAGCACGATTCTACTTAAGCCCTTTCCTTCCTGGATTTTGAGGGAGAATATCTT |
| | | GCCTCTGTCCTTTAGGTTGACTGGAACATAGAGAACCCCAAAAGATCACGGAGTGG |
| | Mutant | CACCCAGAAAAAGGAGGGCTCCTTATTTCCGGATCCTAGAGAGGGG |
| chr | | AACCAACGGATCCCCCAAACTTAGCTATTTGGAAGAAAAAACACAAGATTTAAGGTA |
| 14: | | ATCTGTTGTTAAATGTTATTTGGATACACTAACATCGTGCATGAAAATAAAACTTGTG |
| 380 | | TAAGTGGCTACTTTAAGAAACGCTTTACCTTTCCAAATCAATTCTATTTATAAACAGG |
| 559 | | AAGATTGTGAAATATTCATGTTTTTTTCATTTCATTTTCTTACATTCTTACTCTCATGAG |
| 08 | | AATCTATTGATTCTAACGATTCTTTCAGTTTGAGAAAATTTTGTTTTTAAATTTCAGCA |
| (T> | Wild | TAATTTGATTACCTATTTAGTTGGATCCTAGAGAGGGG |
| C) | Type | AACCAACGGATCCCCCAAACTTAGCTATTTGGAAGAAAAAACACAAGATTTAAGGTA |
| | | ATCTGTTGTTAAATGTTATTTGGATACACTAACATCGTGCATGAAAATAAAACTTGTG |
| | | TAAGTGGCTACTTTAAGAAACGCTTTACCTTTCCAAATCAATTCTATTCATAAACAGG |
| | | AAGATTGTGAAATATTCATGTTTTTTTCATTTCATTTTCTTACATTCTTACTCTCATGAG |
| | | AATCTATTGATTCTAACGATTCTTTCAGTTTGAGAAAATTTTGTTTTTAAATTTCAGCA |
| | Mutant | TAATTTGATTACCTATTTAGTTGGATCCTAGAGAGGGG |
| chr | | AACCAACGGATCCTTTAGTTTTTGCTGAATAGAACTGGTGAGCTTTTCTTCTTCCCTA |
| 14: | | TCTTTGGTTCTGTTTTTGTCACCTTAAAAATGTTTCATACCTGCCCTTCCTAAATGCAA |
| 380 | | GGTGAGAGTAACAATTGCAGCTCCCCTCCCCTGTCCTATGCTCACTCCCAAAACATT |
| 569 | | TGTTTTTTTCTTTTTTGTAAAAATGTTTGCTCGGTAGCGTTGGTGGGTCCGAGCGCCA |
| 77 | | CCGGAGCTGTACACTTGGGTCAGGAGGAAGGCTTTCCCTCCTCGCCCCTCTTCGCCC |
| (T> | Wild | CCTCCCTCCCCTCCCCGGGGACCCGGATCCTAGAGAGGGG |
| G) | Type | AACCAACGGATCCTTTAGTTTTTGCTGAATAGAACTGGTGAGCTTTTCTTCTTCCCTA |
| | | TCTTTGGTTCTGTTTTTGTCACCTTAAAAATGTTTCATACCTGCCCTTCCTAAATGCAA |
| | | GGTGAGAGTAACAATTGCAGCTCCCCTCCCCTGTCCTATGCTCACGCCCAAAACATT |
| | | TGTTTTTTTCTTTTTTGTAAAAATGTTTGCTCGGTAGCGTTGGTGGGTCCGAGCGCCA |
| | | CCGGAGCTGTACACTTGGGTCAGGAGGAAGGCTTTCCCTCCTCGCCCCTCTTCGCCC |
| | Mutant | CCTCCCTCCCCTCCCCGGGGACCCGGATCCTAGAGAGGGG |
| chr | | AACCAACGGATCCATTTTAGAAAAGCCCTGGGTAGCTGGGAGCTGAATAATGTACA |
| 14: | | CACATGGATGTAGTGTATGGAATGACAGACAGTGGAGACTTGGCAGGGTGAGAGG |
| 381 | | GTTGGCAGGAAATGGAGGATAAGAAATTACTTAATGAGTACATTGGGTTATTTGGG |
| 278 | | TGAAAGATACCTTAAAAGCCTTGACTTCTACACAATCTATGCACATAGCAAAAACTAC |
| 42 | | ATTTACACACCATACATTTAAACAAGAAAGAAAAGAAAAGTCCTGGGTAAGTAAAAT |
| (T> | Wild | GTCTGAAAAAGCCTTTAAAAATTTTTTTATTTGGATCCTAGAGAGGGG |
| C) | Type | |

AACCAACGGATCCATTTTAGAAAAGCCCTGGGTAGCTGGGAGCTGAATAATGTACA
CACATGGATGTAGTGTATGGAATGACAGACAGTGGAGACTTGGCAGGGTGAGAGG
GTTGGCAGGAAATGGAGGATAAGAAATTACTTAATGAGTACATTGGGTTATT**C**GGG
TGAAAGATACCTTAAAAGCCTTGACTTCTACACAATCTATGCACATAGCAAAAACTAC
ATTTACACACCATACATTTAAACAAGAAAGAAAAGAAAAGTCCTGGGTAAGTAAAAT
Mutant GTCTGAAAAAGCCTTTAAAAATTTTTTTATTTGGATCCTAGAGAGGGG
